# The role of hyperthermia in the treatment of locally advanced cervical cancer: a comprehensive review

**DOI:** 10.1136/ijgc-2021-002473

**Published:** 2022-01-19

**Authors:** Marloes IJff, Johannes Crezee, Arlene L Oei, Lukas J A Stalpers, Henrike Westerveld

**Affiliations:** 1 Department of Radiation Oncology, Amsterdam University Medical Centers, Cancer Center Amsterdam, University of Amsterdam, Amsterdam, The Netherlands; 2 Laboratory for Experimental Oncology and Radiobiology (LEXOR), Cancer Center Amsterdam, University of Amsterdam, Amsterdam, The Netherlands

**Keywords:** cervical cancer, radiotherapy

## Abstract

Radiotherapy with cisplatin (chemoradiation) is the standard treatment for women with locally advanced cervical cancer. Radiotherapy with deep hyperthermia (thermoradiation) is a well established alternative, but is rarely offered as an alternative to chemoradiation, particularly for patients in whom cisplatin is contraindicated. The scope of this review is to provide an overview of the biological rationale of hyperthermia treatment delivery, including patient workflow, and the clinical effectiveness of hyperthermia as a radiosensitizer in the treatment of cervical cancer. Hyperthermia is especially effective in hypoxic and nutrient deprived areas of the tumor where radiotherapy is less effective. Its radiosensitizing effectiveness depends on the temperature level, duration of treatment, and the time interval between radiotherapy and hyperthermia. High quality hyperthermia treatment requires an experienced team, adequate online adaptive treatment planning, and is preferably performed using a phased array radiative locoregional hyperthermia device to achieve the optimal thermal dose effect. Hyperthermia is well tolerated and generally leads to only mild toxicity, such as patient discomfort. Patients in whom cisplatin is contraindicated should therefore be referred to a hyperthermia center for thermoradiation.

## Introduction

Cervical cancer is the fourth most common cancer in women worldwide, especially in underdeveloped countries,^1^ with approximately 570 000 new cases of cervical cancer and more than 300 000 deaths from this malignancy in 2018.^1^ Cervical cancer most often arises from a persistent infection with the cancer causing human papillomavirus types 16 and 18.^2^ Radiotherapy with cisplatin based chemotherapy as a radiosensitizer (chemoradiation) is the standard treatment for women with locally advanced cervical cancer.^3^ Radiotherapy with deep hyperthermia is a well established alternative. Hyperthermia is a technique that already gained interest in the field of medicine in 1898 by Frans Westermark; he was the first physician to use local tumor heating to treat cervical cancer, by circulating heated water through a metal coil.^4^ His work was carried on by his son, Nils Westermark, who hypothesized that tumor tissue would be more heat sensitive than healthy tissue.^4 5^ In the 1930s, radiologist Kristian Overgaard experimented with the combination of hyperthermia and radiotherapy (thermoradiation), and showed better tumor control with thermoradiation compared with radiotherapy alone.^4^ Hyperthermia, defined by local heating of the tumor up to 42°C for approximately 60 min, has been used as an alternative radiosensitizing treatment in women in whom cisplatin is contraindicated for the treatment of gynecologic cancers, such as vaginal and cervical cancer.^6^ Even though deep hyperthermia has been widely accepted as a radiosensitizer, hyperthermia is rarely offered as an alternative to cisplatin. Despite the evidence, carboplatin is most often offered as an alternative to cisplatin, even though there is less evidence that this works equally well.^7^

The aim of this article is to provide an overview of the clinical data of the effectiveness of hyperthermia as a radiosensitizer through deep hyperthermia in cervical cancer patients, the biological rationale supporting its use, and the patient workflow and equipment used.

## Search Strategy and Selection Criteria

A systematic literature search was conducted to obtain an overview of the existing evidence of hyperthermia in the treatment of cervical cancer. The inclusion criteria were: original clinical studies published after 2000, written in English, and a minimum of 40 included patients. In addition, only studies with a curative intent were included. If the same patient cohort was reported in more papers, only the most recent publication was included. Finally, a reference cross check was performed. Searches in PubMed were performed with the following search terms: “((cervical cancer, uterine(MeSH Terms)) AND (hyperthermia, induced(MeSH Terms))) or ((cervical cancer, uterine(MeSH Terms)) AND (radiotherapy(MeSH Terms)) AND (hyperthermia, induced(MeSH Terms)) or ((cervical cancer, uterine(MeSH Terms)) AND (radiotherapy(MeSH Terms)) AND (hyperthermia, induced(MeSH Terms)) AND (cisplatin(MeSH Terms))) or (((cervical cancer, uterine(MeSH Terms)) AND (hyperthermia, induced(MeSH Terms)) AND (cisplatin(MeSH Terms))).

## Radiobiological Background

**Figure 1:**
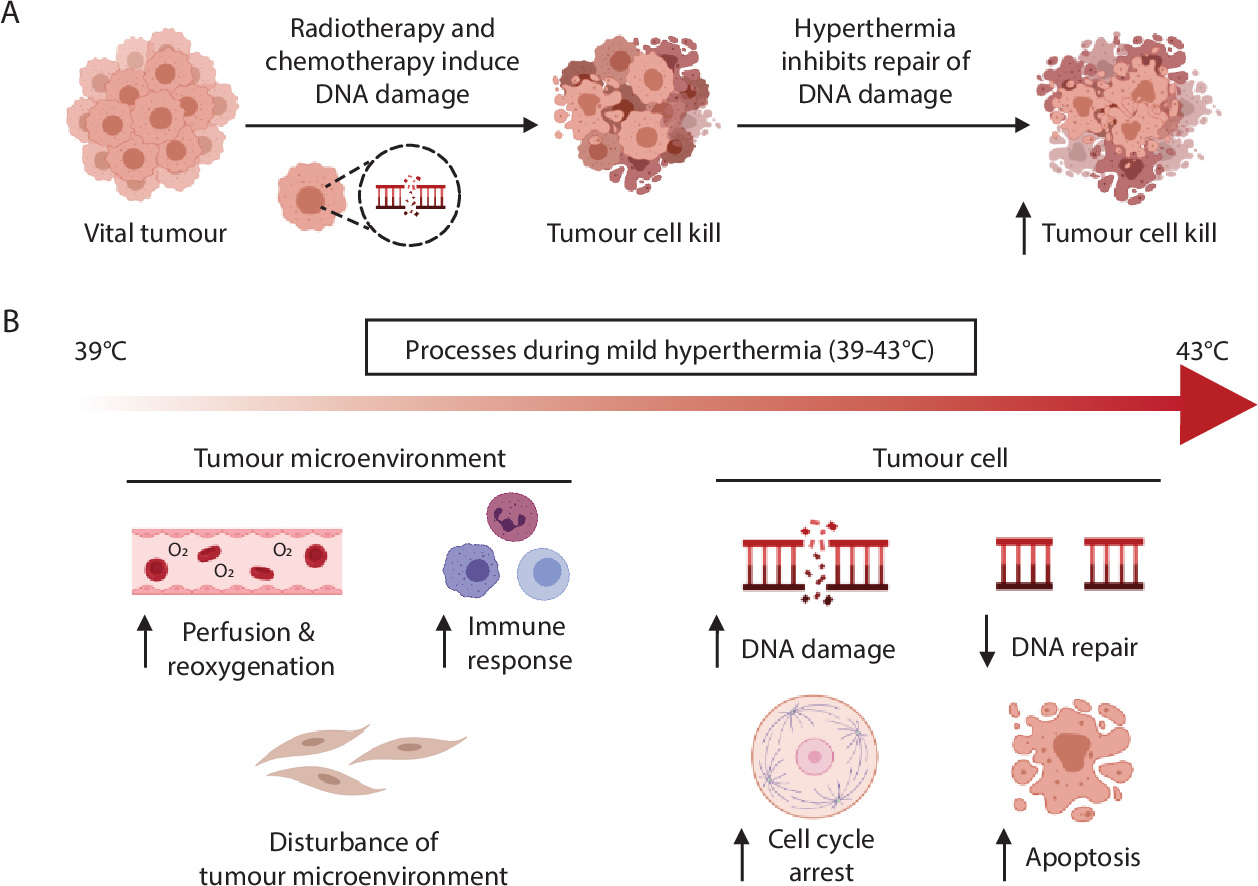
Schematic overview of the enhanced effectiveness of radiotherapy or chemotherapy with hyperthermia. (A) Hyperthermia can temporarily inhibit repair of radiotherapy or chemotherapy induced DNA damage, resulting in increased tumor cell kill. (B) Hyperthermia has effects on both the tumor microenvironment and the tumor cell itself. Already at lower temperatures, starting at 39°C, hyperthermia can disturb the tumor microenvironment by increased perfusion and reoxygenation. Moreover, heat was found to attract immune cells into the tumor microenvironment. Starting at 41°C, hyperthermia can temporarily inhibit DNA repair pathways, resulting in an accumulation of DNA breaks and thereby causing cell cycle arrest. Subsequently, failure to repair DNA breaks causes cell death, such as by apoptosis.

Hyperthermia and cisplatin are potent radiosensitizers. Both are used to increase the cytotoxic effects of ionizing radiation on cancer cells.^7^ Radiotherapy and cisplatin based chemotherapy aim to cause lethal DNA damage, where DNA double strand breaks are considered the most lethal. Ionizing radiation induces DNA double strand breaks directly and indirectly. Induction of DNA breaks immediately triggers DNA double strand break repair pathways.^8^ There are two main DNA double strand break repair pathways: homologous recombination and non-homologous end joining. Hyperthermia can temporarily inhibit DNA repair via the homologous recombination pathway^9^ and the non-homologous end joining pathway,^10^ resulting in accumulation of unrepaired DNA breaks.^11^ The effectiveness of hyperthermia is dependent on the temperature level, duration of treatment, and the time interval between the ionizing radiation and hyperthermia.^12^ Therefore, the combination of ionizing radiation or cisplatin based chemotherapy with adequate hyperthermia treatment (ie, approximately 42°C for 60 min), results in a higher induction of DNA breaks, less DNA repair, and ultimately increased tumor cell death (Figure 1A).^13^ Hyperthermia in combination with chemotherapy is thought to lead to a synergistic effect, rather than an additional effect only.^14^

Hyperthermia contributes to several biological effects on both the tumor and its microenvironment (Figure 1B).^14^ As already known in the 1980s, hypoxic areas in the tumor are radioresistant, and hyperthermia can overcome this resistance by killing these cells directly at higher temperatures.^15^ In addition, increased tumor oxygenation improves the effectiveness of ionizing radiation. Hyperthermia is especially effective in hypoxic and nutrient deprived areas of the tumor where radiotherapy and chemotherapy are less effective. Local hyperthermia improves tumor blood supply, resulting in a decrease in radiation resistance associated with hypoxia.^16 17^ At relatively low temperatures of 39–40°C, perfusion and oxygenation increase, causing changes in pH and thereby altering and affecting the tumor microenvironment. Elevated oxygen levels can enhance the radiation induced DNA breaks and fixate the DNA damage, making it more difficult to repair these breaks. Moreover, hyperthermia was found to attract immune cells into the tumor area.^13 14^ At higher temperatures of up to 43°C, hyperthermia can temporarily inhibit DNA repair pathways, causing cell cycle arrest, that subsequently leads to cell death such as apoptosis (Figure 1B).

As mentioned before, the effectiveness of hyperthermia depends on various factors, including the temperature level, duration of hyperthermia treatment, and the sequence and time interval between radiotherapy and hyperthermia.^12^ Evidence suggests that simultaneous radiotherapy and hyperthermia give the highest enhancement, and the time interval between hyperthermia and ionizing radiation should therefore be kept as short as possible, preferably within 1 hour.^18 19^ Longer intervals will lead to impaired inhibition of DNA repair due to less effectiveness of the hyperthermia, and will consequently lead to increased tumor cell survival.^18^ Even though others found no significant differences within 1–4 hours,^20^ close analysis suggests that the time interval should not exceed 1 hour for full exploitation of the hyperthermia effects.^21^ Some clinical protocols for breast cancer apply nearly simultaneous ultrashort 5 min time intervals between hyperthermia and radiotherapy, however, such short intervals are not feasible in cervical cancer treatment.^22^

## Hyperthermia: Technical Aspects and Patient workflow

### Hyperthermia Devices

The hyperthermia devices currently used for locoregional treatment of deep seated tumors, including cervical cancer, use electromagnetic energy and can be subdivided in two types of systems, radiative and capacitive. Radiative heating devices are phased arrays of 4–12 antennas positioned around the pelvis of the patient, operating at 70–150 MHz.^23^ Capacitive heating devices operate at 8–13 MHz and use two electrodes placed on the ventral and dorsal side of the pelvis. For both devices, a cooled water bolus is placed between the antenna or electrode and the skin to prevent overheating of the skin. Adequate therapeutic tumor temperatures are more easily achieved using radiative devices due to the risk of treatment limiting excessive skin temperatures when using capacitive devices, particularly when the subcutaneous fat layer thickness exceeds ~1 cm.^24 25^ European Quality Assurance guidelines thus recommend use of radiative phased array devices for patients in the Western world.^26^ Hyperthermia treatment delivery requires online temperature monitoring and online adaptation of system settings in response to low tumor temperatures or patient complaints when treatment limiting normal tissue hot spots occur. Online temperature monitoring is performed using minimally invasive temperature probes, typically inserted in the bladder, vagina/cervix, and rectum. Application of non-invasive MRI thermometry is under development for treatment of deep seated pelvic tumors, but patient size and motion artifacts are currently limiting factors for its application and accuracy.^27^ Locoregional heating implies that temperatures in neighboring organs, such as the bladder and rectum, are also raised to elevated levels; this is considered acceptable as hyperthermic radiosensitization is tumor selective and provided temperatures do not exceed 44–45°C. Treatment planning is currently used in select academic centers^26^ where real time (online) adaptive planning is quantitatively reliable.^28^

### Patient Workflow

**Figure 2:**
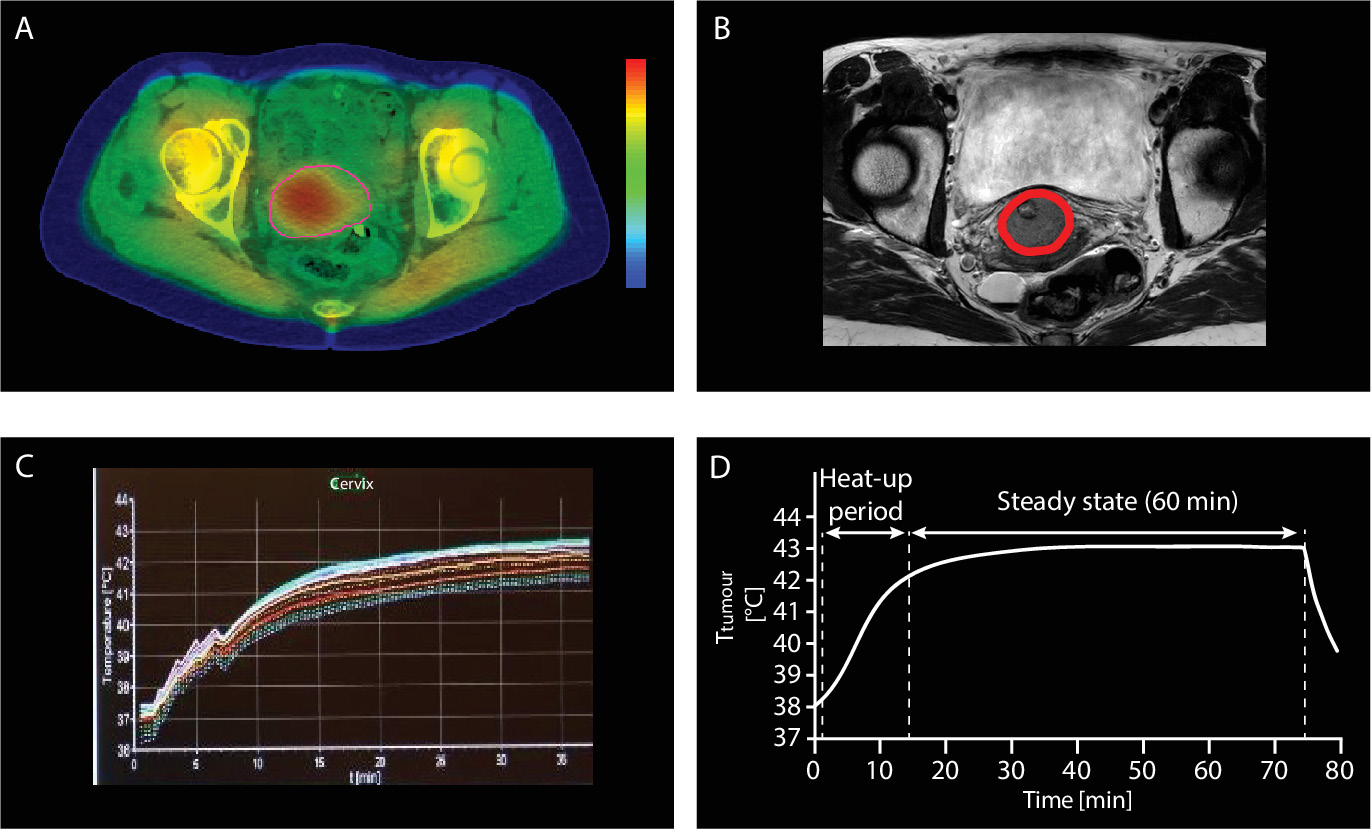
Hyperthermia treatment planning and temperature during treatment. (A) Hyperthermia treatment planning with the cervical tumor contoured in red on a dedicated hyperthermia CT scan with thermal probes in situ made directly before hyperthermia treatment. Also shown are the hot (red area) and cold (green area) spots. (B) MRI scan as help for appropriate contouring of the tumor on CT. (C) Real tumor temperature profile containing temperature readings of target area and surrounding areas during treatment. (D) Simplified tumor temperature profile during treatment.

The workflow for delivery of locoregional hyperthermia treatment involves several steps.^29^ First, hyperthermia should be planned in sequence with radiotherapy delivery. In general, hyperthermia is given once a week shortly before or after the radiotherapy fraction. In some exceptions, hyperthermia is given twice a week, with at least 3 days in between each session to avoid induction of thermotolerance.^30^ To achieve the maximal benefit of hyperthermia as a radiosensitizer, the time interval between radiotherapy and hyperthermia should be less than 1 hour.^18^ In our center, after placement of minimally invasive catheters for insertion of temperature probes in the vagina, bladder, and rectum, a hyperthermia planning CT is made of the patient on the hyperthermia mattress and water bolus around with these thermal probe catheters in situ (Figure 2A). This CT is used for automatic segmentation of high versus low water content tissue for hyperthermia treatment planning, where the tumor is contoured by the physician, guided by the MRI made for radiotherapy planning (Figure 2B). In addition, the CT is used for establishing which temperature measurement points represent tumor and which normal tissue for optimal temperature control during treatment. The aim of the hyperthermia treatment planning is to determine the optimal device settings resulting in good tumor heating while avoiding overheating normal tissues (Figure 2A).^31^

**Figure 3:**
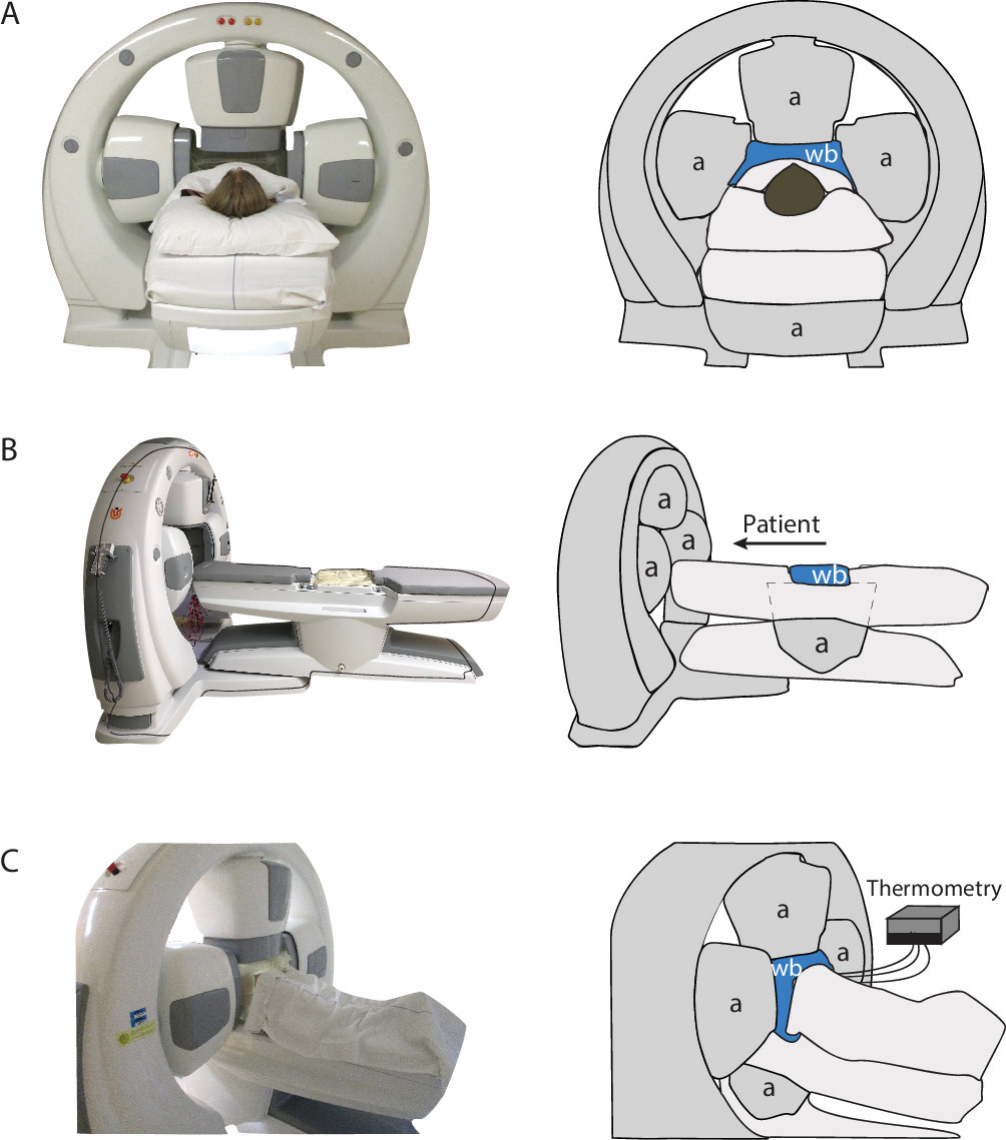
Locoregional radiative hyperthermia device: the example shown here is the four antenna ALBA4D system. (A) Photo and drawing of the front with a patient in position showing the cranial and lateral antennas and the water bolus between the patient and antennas. (B) Photo and drawing from the side, showing that the bottom antenna and a second water cooling bolus is positioned below the patient. (C) Photo and drawing from behind with a patient in position, showing the water cooling boluses on all four sides and the position of the thermometry systems and thermometry probes. a, antenna, wb, water cooling bolus.

After CT, the patient is transported to our deep hyperthermia facility and treatment starts. Multi-sensor temperature probes are inserted into the thermal probes in the vagina, bladder, and rectum. The patient lies on a mattress with four antennas placed around the target volume (Figure 3). To avoid skin burns, water cooling boluses are placed between the antennas and the skin of the patient. Next, the patient is positioned in the system with the tumor at the center of the antenna ring, based on the tumor location on the CT. Minimally invasive temperature monitoring by temperature probes in the cervix, bladder, and rectum is mandatory, and can in selected cases be supplemented with non-invasive MRI based thermometry when using a hybrid locoregional hyperthermia system.^29^ Power is switched on and the heating up period starts (~15–30 min) (Figure 2C, D). When a tumor temperature of 41°C is reached, the 1 hour steady state period starts (Figure 2C, D). Operators continuously monitor the temperature readings and patient comments during treatment, and re-optimize device settings when needed in response to suboptimal tumor temperatures, treatment limiting hot spots, or patients feeling too uncomfortable. This continuous real time monitoring and re-optimization, also guided by adaptive hyperthermia treatment planning, yields optimal tumor temperatures.^28^ However, to be able to deliver such a high quality hyperthermia treatment, an experienced, well trained team is crucial to reach the optimal thermal radiosensitizing effect.^29^ A higher thermal dose can be achieved both by increasing the temperature or by extending the treatment time, where locoregional hyperthermia treatment of 1 hour is considered the maximum patients can tolerate.^32 33^

## Clinical Results Cervix Carcinoma

To obtain an overview of the clinical results of hyperthermia in the treatment of cervical cancer, we conducted a systematic search in PubMed, as described in the search strategy and selection criteria. In total, 365 papers were identified; duplicate and non-English papers were excluded. After screening by two reviewers, 42 papers were included for full evaluation based on the title and abstract. Finally, 10 papers fulfilled the inclusion criteria and will be further discussed. Among these papers, seven were randomized controlled trials and three were cohort studies. The overall patient and treatment characteristics showed that the majority of patients had cervical cancer International Federation of Gynecology and Obstetrics (FIGO) stage II or III. The only exception was in the study of Minnaar et al^34^ in which most patients had FIGO stage IVA disease. All patients were treated with external beam radiotherapy to a total dose of 45–50.4 Gy in 25–28 fractions, followed by a brachytherapy boost. Patients who received chemotherapy usually received weekly cisplatin 40 mg/m^2^. The majority of patients treated with hyperthermia underwent at least four hyperthermia sessions. More details about the selected studies can be found in online supplemental file A.

10.1136/ijgc-2021-002473.supp1 Supplementary data

From the randomized control trials, three studies compared radiotherapy and radiotherapy combined with hyperthermia (thermoradiation),^35 36^ one study compared chemoradiation with thermoradiation,^37^ and three studies compared chemoradiation with chemoradiation combined with hyperthermia^34 38 39^ (Table 1).

**Table 1:** Summary of patient and treatment characteristics, and treatment outcomes of the included randomized controlled trials. Outcome data are expressed at 5 years, unless indicated differently

Author (year of publication)	Years of inclusion	No of patients	Mono/multi center	Treatment arms	Median FU (months)	Age (years)	FIGO stage (n (%))				HT device	HT temp (median °C)	Outcome		
							I	II	III	IV			LC/PC	DFS	OS
Harima (2001)^42^	1994–1999	40	Mono	RT vs RHT	36	62 vs 65	0 (0)	0 (0)	40 (100)	0 (0)	Capacitive	40.6	10 vs 16*	10 vs 16*	48 vs 58*
Van der Zee (2002)^35^	1990–1996	114	Multi	RT vs RHT	43	56 vs 58	0 (0)	22 (19)	81 (71)	11 (10)	Radiative	NA	41 vs 61*	NA	27 vs 51*
Vasanathan (2005)	1998–2002	110	Multi	RT vs RHT	16	50 vs 45	0 (0)	56 (51)	51 (46)	3 (3)	Capacitive	41.6	69*	NA	73*
Lutgens (2016)^37^	2003–2009	84	Multi	CRT vs RHT	85	53	18 (21)	46 (55)	18 (21)	2 (3)	Radiative	NA	NA	1.15†	1.04†
Harima (2016)^38^	2001–2015	101	Multi	CRT vs RCHT	55	62 vs 60	1 (1)	26 (26)	66 (65)	8 (8)	Capacitive	41.1	71 vs 80	61 vs 71	65 vs 78
Minnaar (2019)^34^	2014–2017	202	Mono	CRT vs RCHT	6	49 vs 48	0 (0)	75 (36)	2 (1)	129 (63)	Capacitive	NA	20 vs 39‡	**20 vs 39‡**	82 vs 87‡
Wang (2020)^39^	2009–2013	373	Mono	CRT vs RCHT	60	50 vs 51	7 (2)	230 (62)	127 (34)	9 (2)	Capacitive	40.5	NA	83 vs 87	**72 vs 82**

Bold type indicates significant difference.

*Based on 3 years of follow-up.

†Based on 7 years of follow-up.

‡Based on 6 months of follow-up.

CRT, chemoradiation; DFS, disease free survival; FIGO, International Federation of Gynecology and Obsetrics 2008; FU, follow-up; HT, hyperthermia; LC, local control; NA, not available; OS, overall survival; PC, pelvic control; RCHT, chemoradiation with hyperthermia; RHT, radiotherapy and hyperthermia; RT, radiotherapy.

The older studies, before the introduction of cisplatin as a sensitizer, compared radiotherapy with thermoradiation. In 2000, the results of the Dutch Deep Hyperthermia trial were published.^40^ This randomized, multicenter trial investigated the effect on complete response and persistent local control of radiotherapy versus radiotherapy with hyperthermia in 358 patients with pelvic tumors (bladder, rectal, and cervical cancer). The trial showed a significantly higher complete response rate and better local control in patients treated with the combination of radiotherapy and hyperthermia versus radiotherapy alone. It seemed, however, that the strongest effect was seen in patients with cervical cancer. Therefore, a sub-analysis in the cervical cancer group was performed and published 2 years later.^35^ In this sub-cohort, the 3 year local control rate was 61% versus 41%, and overall survival was 51% versus 27%, respectively, in favor of the thermoradiation group. Notably, the majority (62%) of patients had FIGO stage III disease (43). The combined treatment was well tolerated and no additional hyperthermia related toxicity was seen in the thermoradiation group.^41^

Another randomized trial with 40 patients also showed a significantly better complete response rate (80% vs 50%) in the thermoradiation versus the radiotherapy alone group.^42^ In addition, a trend towards a better disease free survival (64% vs 45%) and overall survival (58% vs 48%) in the thermoradiation group was shown. However, this difference was not statistically significant, probably due to the small sample size. A study by Vasanthan et al, published in 2005, showed no benefit from thermoradiation versus radiotherapy alone.^36^ In this study, there was also no significant difference seen in severe (grade 3) acute and late toxicity. A comment on this multicenter study was that inadequate hyperthermia techniques and quality assurance were applied, and that the reported temperatures overestimated the tumor temperature achieved.^43^

Three randomized trials comparing radiotherapy with thermoradiation were not found by our search because the results were published in non-English journals. Data from these studies were, however, included in a Cochrane review about the combined use of hyperthermia and radiotherapy in locally advanced cervical cancer patients.^44^ This review included six randomized studies published from 1987 to 2009, and showed better outcomes with the addition of hyperthermia to radiotherapy.^44^ Pooled data analysis showed a significantly higher local response rate, and better 3 year local control and overall survival. No differences were seen in acute and late severe toxicity. Notably, 74% of the included patients had FIGO stage III disease.

Only one randomized trial compared chemoradiation with thermoradiation in women with bulky and/or FIGO stage ≥III cervical cancer.^37^ This study was prematurely closed due to a lack of accrual. In total, 84 patients were enrolled.^37^ No significant differences in disease free survival and overall survival between the two treatment arms were found. Although the study was prematurely closed, these results suggest that thermoradiation yields clinical outcomes comparable with outcomes of chemoradiation in the treatment of locally advanced cervical cancer.

Recently, the results of three randomized controlled trials comparing chemoradiation with chemoradiation in combination with hyperthermia were published. The first study from Harima et al (2016) described the results of a multicenter study of 101 patients.^42^ Although no significant differences in disease free survival, overall survival, or complete response were seen, the triple therapy arm performed consistently better than the chemoradiation arm with a gain of all outcome parameters of approximately 10%. The relatively small sample size combined with the fact that some of the patients received a low suboptimal hyperthermia dose, likely explains the non-significant difference. More detailed analysis by Ohguri et al (2018) showed that 5 year disease free survival was 81% for patients in whom a high thermal dose was achieved (CEM43T90 ≥1 min) compared with 61% for patients receiving chemoradiation alone (p=0.036).^25^

A much larger randomized controlled trial of 435 patients showed significantly better overall survival in the triple therapy arm; 5 year overall survival was 82% and 72% for chemoradiation with hyperthermia and chemoradiation, respectively.^38 39^ The difference in relapse free survival was not significantly different. Again, no difference in acute and late toxicity was seen. Finally, Minnaar et al (2019) published the preliminary results of their randomized trial in which 271 patients were included.^34^ They showed a significant benefit of adding hyperthermia to chemoradiation regarding disease free survival, but not overall survival. This might be due to the short median follow-up period of 6 months.^34^ Notably, the results of chemoradiation arm in this study appear to be worse than expected according to current standards. This is probably because the study reports the results of cervical cancer care for advanced stage patients with a relatively poor health status in a low income country with limited resources to treat patients according to best practice standards with external beam radiation therapy combined with chemotherapy, followed by a brachytherapy boost.

A recent meta-analysis concluded that there was a significant benefit of adding hyperthermia to chemoradiation for overall survival, but not for local recurrence free survival. Reassuringly, no increase in toxicity was seen with the addition of hyperthermia.^45^ The previously mentioned chemoradiation with hyperthermia studies all used the easier applicable capacitive hyperthermia device, however, with the cost that it is more challenging to achieve the desired tumor temperature levels. The relevance of an optimal thermal dose was corroborated by a re-analysis of the previously mentioned study by Harima et al (2001). Ohguri et al (2018) found that disease free survival was only better in patients in whom a higher thermal dose was achieved (CEM43T90 >1 min) compared with patients receiving chemoradiation alone.^25^ Although triple chemoradiation with hyperthermia therapy may have additional value over chemoradiation, it is presently not considered as standard of care in the treatment of locally advanced cervical cancer. Interestingly, a recent network analysis identified radiotherapy and hyperthermia, chemoradiation with hyperthermia, and chemoradiation with 3 weekly cisplatin as the best therapeutic modalities for the treatment of locally advanced cervical cancer, comprehensively meeting key clinical endpoints regarding tumor control, survival, and morbidity.^46^ This should, however, be subject to further research because the current standard chemotherapy regimen is with weekly cisplatin 40 mg/m^2^.

The results of the three cohort studies included in our review are summarized in Table 2. Two cohort studies were retrospective in nature, while one study was prospective. Franckena et al (2009) investigated the relationship between thermal dose parameters and the outcomes disease specific survival, pelvic control, and complete response rate. They showed that two different thermal dose parameters both reflecting median tumor temperature and duration of heating, TRISE (p=0.002 for disease specific survival; p=0.021 for pelvic control; and p=0.027 for complete response), and CEM43T90 (p=0.001 for disease specific survival; p=0.019 for pelvic control; and p=0.195 for complete response) were independent prognostic factors for tumor control.^47^ The association between median thermal dose and outcome was confirmed in a later study by Kroesen et al.^32^ They showed that thermal dose also had a beneficial effect on local control in patients treated according to the current standards with external beam radiation therapy followed by MRI guided brachytherapy.^32^

Finally, Westermann et al (2012) published the long term results of triple therapy (chemoradiation with hyperthermia) in locally advanced cervical cancer patients and concluded that this combination of therapy is feasible, well tolerated, and comparable with the results of randomized trials at that time. However, since it was a non-randomized study, no further conclusions could be drawn.^48^ Carboplatin monotherapy is often offered as an alternative radiosensitizer to cisplatin in the treatment of locally advanced cervical cancer, even though there is no evidence that this works equally well. Moreover, no clinical trials comparing hyperthermia and radiotherapy with carboplatin and radiotherapy have been conducted or planned. However, a few small clinical studies investigated the effect of combining carboplatin monotherapy with hyperthermia and radiotherapy.^49 50^ One phase I study used a combination of radiotherapy, hyperthermia, and intra-arterial carboplatin in 15 cervical cancer patients with a local recurrence.^49^ Although this regimen was well tolerated, the results were disappointing. Another phase II study evaluated the effect of whole body hyperthermia in combination with carboplatin in 25 patients with recurrent or metastatic cervical cancer.^50^ Considerable toxicity was seen and the results were comparable with chemotherapy only, and thus this regimen was considered as less suitable in these palliative patients.

**Table 2:** Summary of patient and treatment characteristics and treatment outcomes of the included cohort studies. Outcome data are expressed at 5 years, unless indicated differently

Author (year of publication)	Years of inclusion	No of patients	Mono/ multi center	Prospective/ retrospective	Median FU (months)	Age (years)	FIGO stage (n (%))				HT device	HT temp (median °C)	Outcome		
							I	II	III	IV			LC/PC	DSS	OS
Franckena (2009)^47^	1996–2005	378	Multi	Retrospective	44	58	13 (3)	160 (42)	163 (43)	42 (11)	Radiative	40.6	53	47	40
Westermann (2012)^48^	1998–2002	68	Multi	Prospective	81	45	3 (4)	42 (62)	21 (31)	2 (3)	Radiative	40.7	NA	58	66
Kroesen (2019)^20^	2005–2016	227	Mono	Retrospective	52	54	32 (14)	118 (52)	53 (23)	24 (11)	Radiative	40.5	73	60*	40*

*Based on 12 years of follow-up.

DFS, disease free survival; FIGO, International Federation of Gynecology and Obstetrics stage 2008; FU, follow-up; LC, local control; NA, not available; OS, overall survival; PC, pelvic control.

Some studies only reported the intended temperature level without measuring temperatures to verify whether the goal temperature was actually achieved. The fact that two different types of hyperthermia systems were used (capacitive and radiative) may also have influenced outcome, because achieving the targeted temperature is more challenging for capacitive devices. All of the included studies in Europe used radiative hyperthermia systems, while many non-European studies used capacitive hyperthermia systems.

Good hyperthermia treatment delivery requires a team of well trained and experienced professionals, dedicated treatment protocols, reliable hyperthermia devices, and treatment planning and quality assurance. Lack of these has been a cause for failure in some clinical trials and an impediment for wider clinical use of hyperthermia. In the past decade, hyperthermia systems have improved and guidelines have been developed.^23 26 29^ Fortunately, reliable treatment planning tools enabling real time adaptive planning are becoming available.^28^ A group of European centers are developing multicenter prospective registration studies with well designed quality assurance and data reporting for several tumor sites in the framework of the European H2020 ‘Hyperboost’ project (www.hyperboost.eu). These developments will help new users to more easily adopt and apply clinical hyperthermia.

In the studies included, toxicity was generally graded according to the toxicity criteria for adverse events. Although no additional severe toxicity was seen in the hyperthermia trials, hyperthermia can lead to acute and late toxicities in some cases. Thermal burns and fat necrosis in particular are considered hyperthermia related toxicities, and can be burdensome for the patient.^35^ In addition, myopathies and patient discomfort can be seen during and shortly after a hyperthermia session.^37^ The risks of hyperthermia related toxicities can, however, be limited when following good quality assurance protocols. Although currently patient reported outcome measures are often used to assess the burden of the treatment on quality of life, no patient reported outcome measures were assessed in the previously mentioned hyperthermia trials. Expert opinion is that thermoradiation is more tolerable than chemoradiation and could therefore be offered to fragile patients who are unfit for chemotherapy. The typical patients referred for hyperthermia are those who have contraindications to cisplatin. These include women with poor kidney function and hearing loss, but also the elderly and frail patients. In addition, patients who refuse chemotherapy can also be referred for hyperthermia treatment. The only contraindications for deep hyperthermia are metal hip prostheses and pacemakers,^29^ making this treatment combined with radiotherapy suitable for most patients with locally advanced cervical cancer.

## Future Perspectives and Conclusions

Radiative locoregional hyperthermia devices are currently optimal for achieving therapeutic temperatures in deep seated tumors, such as in cervical cancer. A novel approach based on induction of hyperthermia by scanning a high intensity focused ultrasound beam through the tumor volume is under development, but its use in humans needs to be tested.^51^ Reliable hyperthermia delivery also requires real time temperature monitoring using minimally invasive temperature probes inserted in the vagina/cervix, bladder, and rectum. Non-invasive MRI based thermometry is under development, but its accuracy is presently still strongly limited by motion artifacts, and about half of the patients do not fit into the small bore of the hybrid MRI guided locoregional hyperthermia device.

Pretreatment planning is another valuable tool for optimizing treatment delivery. Treatment planning is currently qualitatively reliable and able to establish system settings reliably targeting the tumor. Real time online adaptive planning during treatment is key in re-optimizing settings during hyperthermia treatment for optimal tumor temperatures and suppression of potential treatment limiting normal tissue hot spots. Commercially available adaptive treatment planning software is under development, including VEDO^52^ and Plan2Heat,^53^ which allow planning based real time steering during treatment. These tools allow novice hyperthermia users to quickly gain good treatment control. Immunotherapy combined with chemotherapy and radiotherapy is increasingly investigated in cervical cancer trials, and has especially been explored in high risk, locally advanced and metastatic cervical cancer.^54^ Although immunotherapy might have a synergistic effect when combined with hyperthermia, so far no clinical studies have been performed or have been planned that combine immunotherapy with hyperthermia and radiotherapy.

In conclusion, cisplatin combined with radiotherapy is the current standard treatment for patients with locally advanced cervical cancer. However, thermoradiation is the best evidence based, well tolerated alternative and should be offered to all patients with contraindications to cisplatin.
