# Supplementary material for: The role of hyperthermia in the treatment of locally advanced cervical cancer: a comprehensive review
Source: Int J Gynecol Cancer. 2022 Jan 19;32(3):288–96. doi: 10.1136/ijgc-2021-002473 (PMC8921566; doi:10.1136/ijgc-2021-002473)
Supplement: Supplementary data [file ijgc-2021-002473supp001.pdf]

BMJ Publishing Group Limited (BMJ) disclaims all liability and responsibility arising from any reliance placed on this supplemental material which has been supplied by the author(s)

Supplement A. Full overview of the patients and treatment characteristics, and treatment outcomes of the included studies

**Author**	**Journal**	**Year**	**RCT/ cohort**	**Treatment arms**	**Mono/multi center**	**Prospective/ retrospective**	**Median FU (months)**	**Patient numbers**	**Age (years)**	**FIGO Stage**				**Total Dose EBRT**	**Boost brachy**	**type of HT**	**HT sessions ≥4x n (%)**	**Chemotherapy (dose, frequency)**	**Outcome**			**HT-related toxicity ≥grade 3**
										**I**	**II**	**III**	**IV**						**LC/PC**	**DSS/DFS**	**OS**	
Harima	IJH	2001	RCT	RT vs. RHT	Mono	Prospective	36	40	62 vs. 65	0 (0)	0 (0)	40 (100)	0 (0)	52.2 Gy	Yes	Capacitive	3 Sessions		**10 vs. 16***	**10 vs. 16***	48 vs. 58*	2/20 (10) at 3 years; not significant different from RT alone
Van der Zee	IJH	2002	RCT	RT vs. RHT	Multi	Prospective	43	114	56 vs. 58	0 (0)	22 (19)	81 (71)	11 (10)	46-50.4 Gy	Yes	Radiative	40/58 (69)		**41 vs. 61***	N.A.	**27 vs. 51***	No significant difference between treatment groups.
Vasanathan	IJROBP	2005	RCT	RT vs. RHT	Multi	Prospective	16	110	50 vs. 45	0 (0)	56 (51)	51 (46)	3 (3)	50 Gy	Yes	Capacitive	N.A.		69*	N.A.	73*	Acute tox grade 3: 1x blister 1/55 (2); Late tox grade 3: 2x bowel 2/55 (4)
Lutgens	RO	2016	RCT	CRT vs. RHT	Multi	Prospective	85	84	53	18 (21)	46 (55)	18 (21)	2 (3)	50 Gy	Yes	Radiative	38/42 (90)	weekly cispl 40mg/m2	N.A.	1.15^	1.04^	No significant difference between treatment groups.
Harima	IJH	2016	RCT	CRT vs. RCHT	Multi	Prospective	55	101	62 vs. 60	1 (1)	26 (26)	66 (65)	8 (8)		Yes	Capacitive	47/51 (92)	weekly cispl 30-40 mg/m2	71 vs. 80	61 vs. 71	65 vs. 78	No hyperthermia related toxicity was observed
Minnaar	Plos One	2019	RCT	CRT vs. RCHT	Mono	Prospective	6	202	49 vs. 48	0 (0)	75 (36)	2 (1)	129 (63)	50 Gy	Yes	Capacitive	N.A.	2x cispl 80mg/m2 during EBRT in 21 days	20 vs. 39†	**20 vs. 39†**	82 vs. 87†	No significant difference between treatment groups.
Wang	IJROBP	2020	RCT	CRT vs. RCHT	Mono	Prospective	60	373	50 vs. 51	7 (2)	230 (62)	127 (34)	9 (2)	50.4 Gy	Yes	Capacitive	175/182 (96)	cispl 30 mg/m2, d1-3; 5-fluorouracil 350 mg/m2, d1-5	N.A.	83 vs. 87	**72 vs. 82**	No significant difference between treatment groups.
Franckena	IJROBP	2009	Cohort		Multi	Retrospective	44	378	58	13 (3)	160 (42)	163 (43)	42 (11)	46-50.4 Gy	Yes	Radiative	339/378 (90)		53	47	40	45/378 (12) at 5 years; No significant different from RT alone
Westermann	IJH	2012	Cohort		Multi	Prospective	81	68	45	3 (4)	42 (62)	21 (31)	2 (3)	45-50.4 Gy	Yes	Radiative	63/68 (93)	weekly cispl 40mg/m2	20 vs. 39	**20 vs. 39**	82 vs. 87	No significant difference between treatment groups.
Kroesen	OBGNAS	2019	Cohort		Mono	Retrospective	52	227	54	32 (14)	118 (52)	53 (23)	24 (11)	46-50.4 Gy	Yes	Radiative	219/227 (96)		73	60^^	40^^	No significant difference between treatment groups.

FIGO: International Federation of Gynecology and Obsetrics stage 2008; RCT: randomized control trial; RT: radiotherapy; RHT: radiotherapy and hyperthermia; CRT: chemoradiation; RCHT: chemoradiation with hyperthermia; FU: follow-up; LC: local control; PC: pelvic control; DFS: disease free survival; DSS: disease specific survival; OS: overall survival; cispl: cisplatin; N.A.: not available; vs.: versus; *: based on 3-year follow up; ^: based on 7-years follow-up; †: based on 6 months follow up; ^^: based on 12-years follow-up; bold: significant different;
